# Family Physician Perspectives on Primary Immunodeficiency Diseases

**DOI:** 10.3389/fmed.2016.00012

**Published:** 2016-03-30

**Authors:** Jordan S. Orange, Filiz O. Seeborg, Marcia Boyle, Christopher Scalchunes, Vivian Hernandez-Trujillo

**Affiliations:** ^1^Section of Immunology, Allergy and Rheumatology, Department of Pediatrics, Texas Children’s Hospital, Baylor College of Medicine, Houston, TX, USA; ^2^Immune Deficiency Foundation, Towson, MD, USA; ^3^Department of Pediatrics, Division of Allergy and Immunology, Miami Children’s Hospital, Miami, FL, USA

**Keywords:** primary immunodeficiency disease, family practice physicians, survey, diagnosis, treatment

## Abstract

Primary immunodeficiency diseases (PIDs) include over 250 diverse disorders. The current study assessed management of PID by family practice physicians. The American Academy of Allergy, Asthma, and Immunology Primary Immunodeficiency Committee and the Immune Deficiency Foundation conducted an incentivized mail survey of family practice physician members of the American Medical Association and the American Osteopathic Association in direct patient care. Responses were compared with subspecialist immunologist responses from a similar survey. Surveys were returned by 528 (of 4500 surveys mailed) family practice physicians, of whom 44% reported following ≥1 patient with PID. Selective immunoglobulin A deficiency (21%) and chronic granulomatous disease (11%) were most common and were followed by significantly more subspecialist immunologists (*P* < 0.05). Use of intravenously administered immunoglobulin and live viral vaccinations across PID was significantly different (*P* < 0.05). Few family practice physicians were aware of professional guidelines for diagnosis and management of PID (4 vs. 79% of subspecialist immunologists, *P* < 0.05). Family practice physicians will likely encounter patients with PID diagnoses during their career. Differences in how family practice physicians and subspecialist immunologists manage patients with PID underscore areas where improved educational and training initiatives may benefit patient care.

## Introduction

Primary immunodeficiency diseases (PIDs) comprise more than 250 heterogeneous disorders (1). These diseases are caused by intrinsic defects in the immune system that lead to aberrant immune responses (2). The field of PID has evolved, and the number of recognized disorders has rapidly increased (3–5), creating challenges for maintaining up-to-date disease classifications and for disseminating practical patient management guidelines.

A recent analysis of registry data and epidemiologic surveys estimated that as many as six million people worldwide are living with PID, with the majority undiagnosed (27,000 patients identified from national registries; 60,000 patients identified from the Jeffrey Modell Centers Network) (3). A prior household random-digit dialing telephone survey indicated that as many as 150,000–360,000 persons in the United States have been diagnosed with a PID (6). Precise data on prevalence are unavailable at least in part because of the heterogeneity of this group of diseases, lack of newborn screening (4, 6), and the growing number of recognized immunodeficiency diseases (3–5). The 2007 telephone survey indicated that these diseases impact an estimated 1 in 1200 United States persons (6). These data suggest that PID prevalence is likely underestimated, indicating most physicians are likely to encounter patients with PID over the duration of their practicing careers. Expanded appreciation of the prevalence of these diseases (6–9) has been accompanied by improved recognition and diagnostic testing (10–12), particularly with ongoing initiatives to promote recognition and guidelines for the care of patients with PID (1, 13–17).

Early recognition and diagnosis of PID is important for patient prognosis and quality of life (18). However, PID is generally not detected until after a patient has experienced repeated infections. Currently, the time between onset of symptoms and treatment may be as long as 12 years (19). During this intervening period, recurrent infections and improperly managed disease may lead to morbidity and mortality and upper and lower airway infections that may cause pulmonary complications and eventual terminal lung disease (18, 20–22). Therefore, the ultimate goal is to identify patients with PID prior to the onset of clinical symptoms.

Primary care physicians (PCPs) are usually the front-line health-care providers with whom patients first consult with health issues and are therefore the first physicians to treat infections and other symptoms arising in patients with undiagnosed PID. Earlier recognition of signs of an underlying PID may lead to earlier referral to an immunologist, reducing time to diagnosis and initiation of treatment. However, the recognition of cases within such diverse and numerous subclassifications of disease can be a challenge for physicians who do not specialize in immunology. Family practice physicians comprise a primary care specialty that provides comprehensive care to both adult and pediatric patients. Further understanding of the medical training and practice among family practice physicians with regards to the diagnosis and treatment of PID may reveal areas for educational initiatives to improve awareness and ultimately identification of patients with the disease.

Joint efforts of the American Academy of Allergy, Asthma, and Immunology (AAAAI) Primary Immunodeficiency Committee and the Immune Deficiency Foundation (IDF) have allowed for numerous surveys to gain understanding of how physicians diagnose, manage, and treat patients with PID (23–26). These have provided insights into typical clinical approaches in an area that has historically been the domain of a small number of leading experts.

The primary goal of the current survey was to gain insight into the practice of family practice physicians in order to identify opportunities for improvements regarding the diagnosis and management of patients with PID likely encountered over the course of their clinical practice. The objectives of the current study were to report family practice physician responses to the PID survey and to compare them with responses from a previous survey of subspecialist immunologists.

## Materials and Methods

### Survey Subjects

We conducted a survey among members of the American Medical Association and the American Osteopathic Association. Family practice physicians in direct patient care were eligible to take part in the survey and were mailed a four-page questionnaire.

At the time of this study, the team did not seek a full ethics, Internal Review Board (IRB) determination for need for oversight. However, this study does meet the criteria listed in 45 CFR 46.101(b), Category: research involving the use of educational tests, survey procedures, interview procedures, or observation of public behavior, and hence would be exempt for the need for IRB oversight.

### Survey Design and Administration

The survey questionnaire (http://primaryimmune.org/wp-content/uploads/2015/01/Family-Practice_qx.pdf) was originally developed through collaboration of the AAAAI Primary Immunodeficiency Committee and the IDF to gain understanding of the scope of PID practice (23). The aim of this study was to use this instrument to evaluate family practice physician understanding related to recognition and diagnosis of PID. The survey collected information on primary patient care settings, number of PID diagnoses followed, awareness of PID identification testing and treatment strategies, use of immunoglobulin (Ig) replacement therapy, hygiene-based interventions, and vaccination recommendations. The four-page, self-administered survey was anonymous.

Physician sampling (names and addresses) was purchased from an American Medical Association Physician Masterfile of physicians identified as family practitioners. The first mailing occurred on September 15, 2009 and the second mailing, which was sent to previous non-responders, was on October 23, 2009. Data collection was completed in January 2010. A US$25 incentive (from the IDF) was offered to physicians who completed the survey.

### Data Analysis

Only physicians who self-identified their specialty as family medicine (including family medicine whether they focus upon children, adults, or both) were included in the analysis. Returned, completed surveys determined the analysis population.

### Statistical/Other Analyses

Survey responses were collected and a descriptive analysis was carried out for all survey questions. For some data sets, family practice physician responses were compared with responses to a previous survey of allergist/immunologist members of the AAAAI who devote >10% of their practice to patients with PID and are therefore considered “subspecialist immunologists” or “focused” immunologists (23). A separate statistical analysis of those responses was carried out using χ^2^ and Fisher’s exact tests. A *P* value of <0.05 was considered significant. Analysis was conducted using PASW Statistics 18 (SPSS Inc., an IBM Company, Chicago, IL, USA).

## Results

### Survey Response

Of the 4500 surveys mailed, 528 completed surveys were returned [14 respondents were excluded from the analysis (5 saw “0” patients on an outpatient basis; 9 failed to answer the question asking how many patients they saw on an outpatient basis)]. A 12% response rate is similar to the rate achieved in a prior survey that assessed PID management among subspecialist allergist/immunologist members of AAAAI (23). Although these response rates may be low, they likely represent the general understanding that PIDs are a rare group of diseases.

### Demographics of Survey Respondents

The majority (89%) of respondents reported a primary specialty of family medicine and 41% reported being a part of a single-specialty group (Table 1). Approximately half (48%) reported graduating from medical school in 1990 or later; the mean year of graduation was 1987. This relatively recent date of graduation for the responding population are unlikely to fully represent the average age of the total population of practicing family practice physicians in the United States. Comparatively, the mean year of graduation among the subspecialist immunologists was 1978.

**Table 1:** **Demographics of respondents**.

Characteristic	Family practice physicians (*N* = 528)	Subspecialist immunologists (*N* = 71)
**Year of graduation**		
≥1990, *n* (%)	246 (48)	18 (25)
1975–1989, *n* (%)	219 (43)	28 (39)
≤1974, *n* (%)	44 (9)	25 (35)
Missing, *n* (%)	5 (1)	0 (0)
Mean	1987	1978
**Training,^a^*n*(%)**		
Family medicine (adults and children)	469 (89)	NA
Family medicine (adults)	33 (6)	NA
Other?^b^	21 (4)	NA
Family medicine, pediatrics	4 (1)	NA
**Primary patient care setting,^c^*n*(%)**		
Single-specialty group	217 (41)	9 (13)
Multispecialty group	102 (19)	6 (8.5)
Solo practice	105 (20)	4 (6)
Hospital outpatient	29 (6)	41 (58)
HMO	12 (2)	0 (0)
Other^d^	60 (12)	7 (10)
**PID coverage in medical school,^e^*n*(%)**		
“Only a little”	399^f^/526 (76)	NA
“Adequately”	63^f^/526 (12)	NA
“Not at all”	61^f^/526 (11)	NA
“Very well”	3^f^/526 (1)	NA

*HMO, health maintenance organization; NA, not applicable; PIDs, primary immunodeficiency diseases*.

*^a^One respondent did not provide information about primary specialty*.

*^b^Answers given for other includes acupuncture, anesthesia residency, care of the developmentally disabled, critical care medicine, deployment medicine (army), emergency medicine, geriatrics, holistic medicine, hospitalist, musculoskeletal medicine, obstetrics–gynecology, occupational medicine, sports medicine, student health, and urgent care*.

*^c^Three respondents did not provide information about primary patient care setting*.

*^d^Answers given for other includes academic outpatient, blank (no response), college/university health center, community health-care clinic (including community outpatient, clinic, and urgent care), corporate primary care (including workers comp), correctional facility, education (including faculty, teaching medical school, residency program, residency clinic, and residency faculty group), emergency medicine, federally funded health center (including federally qualified health center and federally qualified rural health center), free clinics for the poor, health intervention services clinic, health department, health policy, hospital outpatient, locum tenens, military (including veterans affairs clinic, military/federal contractor, veteran’s medical affairs, military base clinic, military outpatient clinic, and Tricare), no ambulatory, no outpatient work, nursing home, psychiatric hospital, skilled nursing facility, small practice (two physicians), state development center, and urgent care clinic*.

^e^Question in survey was “How fully were primary immunodeficiency diseases covered in medical school?”

*^f^*N* = 526*.

Overall, we hypothesized that treatment practices may reflect introduction to PID in medical school or post-medical school training; therefore, we sought to discern the level of coverage. The majority (76%) of family practice physicians reported that PID was covered “only a little” in medical school, with 11% responding that PID was not covered at all and 12% indicating that PID was “adequately” covered (Table 1). Exposure beyond medical school training was limited among family practice physicians in this survey, with 99% reporting that they have not heard a lecture regarding PID identification and diagnosis in the last 6 months, despite more than half (66%) believing such a lecture would be beneficial. The lack of sufficient PID exposure and training in medical school and beyond may indicate a specific inefficiency in education programs related to PID, where significant improvements could be easily achieved.

### Clinical Experience with PID among Respondents

**Figure 1:**
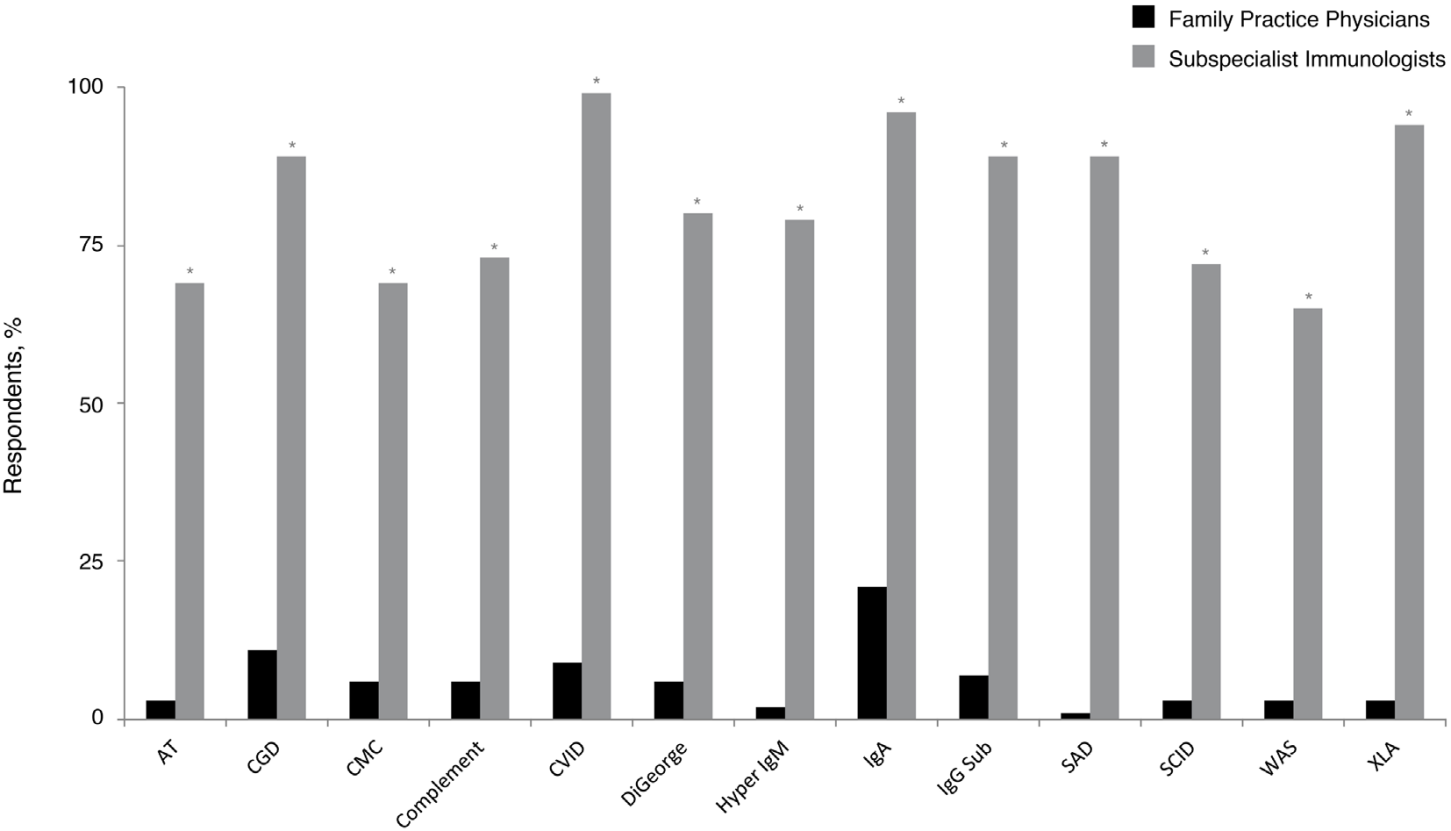
**Percentage of family practice physicians, compared with subspecialist immunologists, who have ever followed patients with a PID**. *P < 0.0001. AT, ataxia telangiectasia; CGD, chronic granulomatous disease; CMC, chronic mucocutaneous candidiasis; CVID, common variable immunodeficiency; IgA, immunoglobulin A; IgG, immunoglobulin G; IgM, immunoglobulin M; PID, primary immunodeficiency disease; SAD, selective antibody deficiency; SCID, severe combined immunodeficiency; WAS, Wiskott–Aldrich syndrome; XLA, X-linked agammaglobulinemia (Bruton’s agammaglobulinemia).

Given the emerging data that indicate PID prevalence is likely underestimated (6–9) and that recent United States population estimates common variable immunodeficiency (CVID) occurs in 1:2400 persons (6), these patients should statistically be present in many PCP practices. In the current survey, 1% of family practice physicians reported following five or more patients with a PID diagnosis, whereas approximately half (56%) reported that they had not followed any. Although CVID is the most commonly occurring PID (6, 21, 27), significantly fewer responding family practice physicians (9%) reported following at least one patient with this diagnosis, compared with 99% of subspecialist immunologists (*P* < 0.05) (Figure 1). Subspecialist immunologists also reported that they had followed patients with diagnoses that were rarely reported as present in family physician practices. Selective immunoglobulin A (IgA) deficiency (21%) and chronic granulomatous disease (CGD) (11%) were the most common diagnoses followed among family practice physicians who reported following at least one patient with PID. As expected, both diagnoses were significantly more prevalent among the practices of subspecialist immunologists (96% IgA and 89% CGD; *P* < 0.05 for each).

### Clinical Management of PID among Respondents

A substantial gap exists in the awareness of professional guidelines for the diagnosis and management of PID (13, 28) between family practice physicians and subspecialist immunologists. Four percent of family practice physicians were aware of these guidelines, compared with 79% of subspecialist immunologists (*P* < 0.05). Although this level of expertise for the treatment of PID is not expected outside the specialty of immunology, this substantial difference underlies the importance of initiatives that strive to promote awareness among physicians who participate in the care of patients with PID.

**Figure 2:**
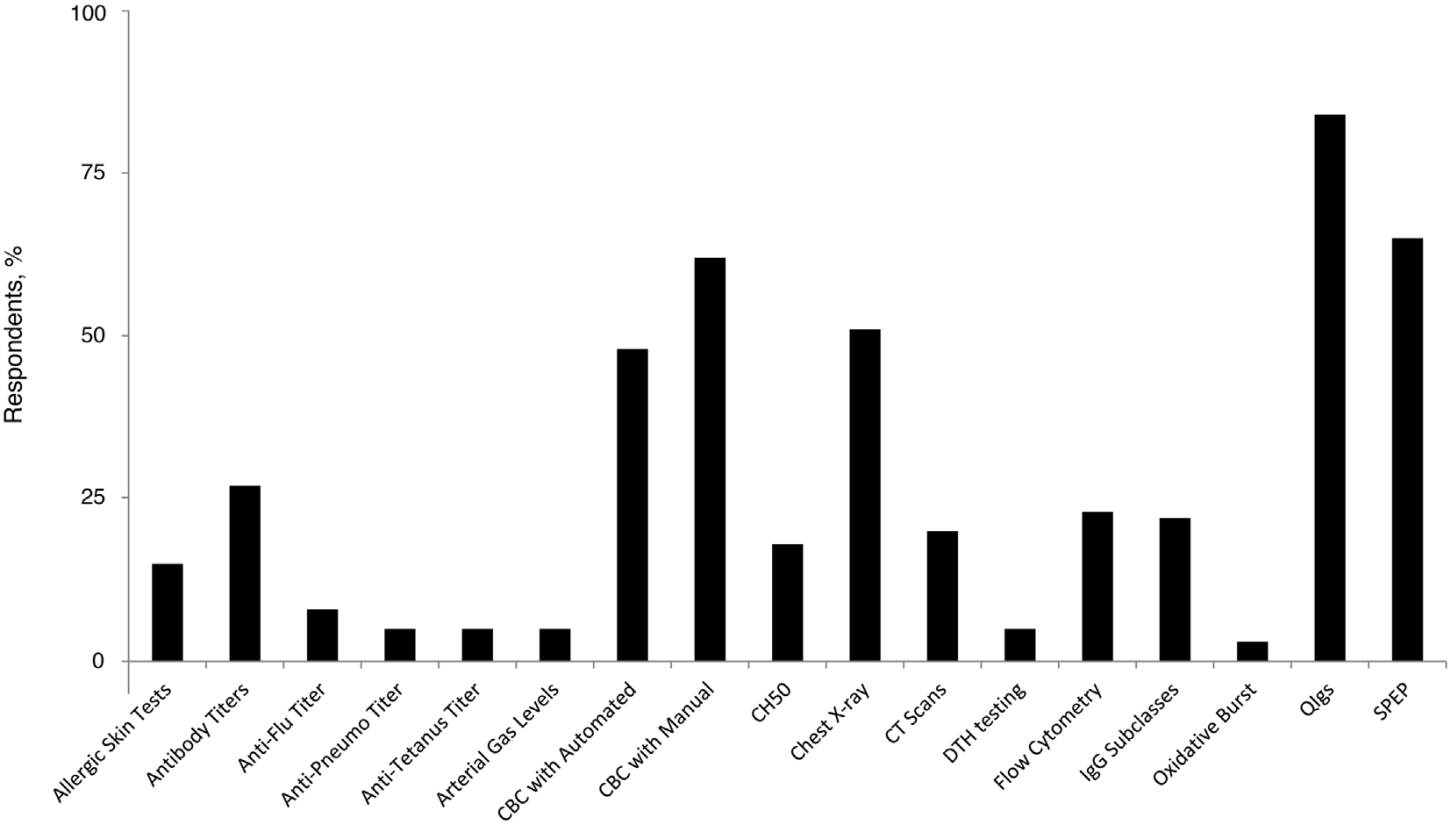
**Percentage of family practice physicians ordering specific tests for the diagnosis of PID**. Anti-Pneumo titer, anti-Pneumococcal titer; CBC, complete blood count; CH50, hemolytic complement; CT, computed tomography; DTH, delayed-type hypersensitivity; IgG, immunoglobulin G; PID, primary immunodeficiency disease; QIgs, quantitative serum; SPEP, serum immunoelectrophoresis.

The majority (77%) of family practice physicians reported that they were not at all comfortable with recognizing and diagnosing PID, and none indicated complete comfort in recognizing and diagnosing among the group of disorders. However, 22% reported being “somewhat” comfortable. Forty percent of family practice physicians reported that if they suspect a patient to have an underlying PID, they will refer the patient to a specialist (including specialists in the field of allergy, immunology, and hematology). Similar proportions will order tests (26%) or will order tests and also refer the patient to a specialist (23%); only 6% reported that they had independently diagnosed a patient with PID. The most commonly ordered diagnostic tests in pursuit of a PID diagnosis among PCP family practice physicians included quantitative serum Ig (84%), serum immunoelectrophoresis (65%), and a complete blood count with manual differential (62%) (Figure 2). Recent recommendations advise against testing for IgG subclasses when PID is suspected (29) and question its clinical relevance (30). However, 22% of family practice physicians still use it as a diagnostic approach, underlying a potential gap in patient management that has gained some primary care focus through the ABIM Choosing Wisely initiative.

### Use of Ig Replacement for PID

**Figure 3:**
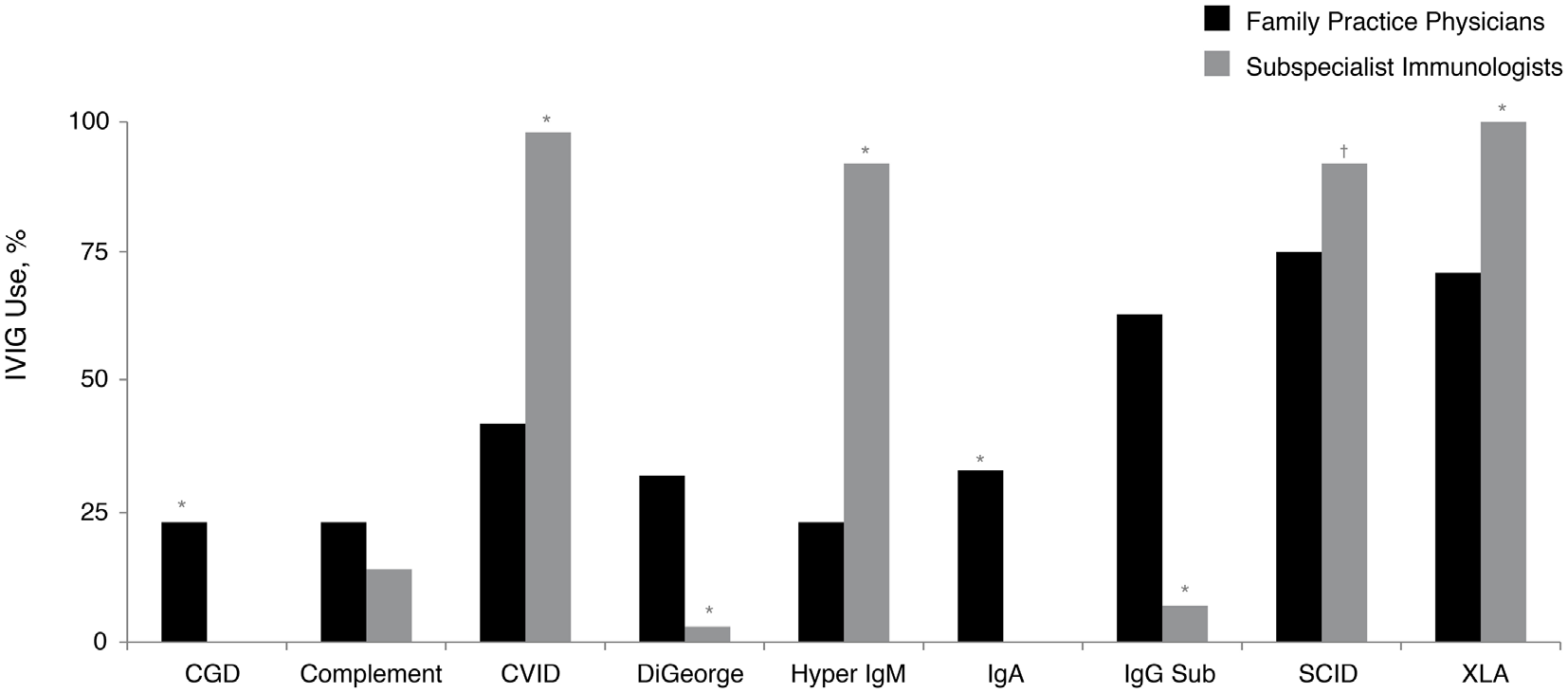
**Percentage of family practice physicians, compared with subspecialist immunologists, recommending IVIG therapy for all or most patients with a diagnosis of a specific PID (in at least some patients within the listed diagnosis)**. *P < 0.0001; ^†^*P* = 0.006. CGD, chronic granulomatous disease; CVID, common variable immunodeficiency; IgA, immunoglobulin A; IgG Sub, immunoglobulin G subclass; IgM, immunoglobulin M; IVIG, intravenously administered immunoglobulin; PID, primary immunodeficiency disease; SCID, severe combined immunodeficiency; XLA, X-linked agammaglobulinemia (Bruton’s agammaglobulinemia).

A small proportion (5%) of family practice physicians reported being the primary treatment provider for patients with PID. Replacement therapy with various intravenously (IV)- and subcutaneously (SC)-administered preparations of Ig (IVIG and SCIG, respectively) is approved by the United States Food and Drug Administration and commonly used for the treatment of patients with PID (28). While nearly all subspecialist immunologists who treat patients with PID commonly recommend IVIG therapy for their patients with Bruton’s agammaglobulinemia (X-linked agammaglobulinemia; XLA), CVID, hyper IgM syndrome (hyper IgM), and severe combined immunodeficiency disease (SCID), significantly fewer family practice physicians recommended IVIG therapy for these same diagnoses: XLA, 100 vs. 71% (*P* < 0.05); CVID, 98 vs. 42% (*P* < 0.05); hyper IgM, 92 vs. 23% (*P* < 0.05), and SCID, 92 vs. 75% (*P* < 0.05) (Figure 3). Furthermore, 65% of prescribing family practice physicians believed that IVIG therapy was only a somewhat-effective treatment approach for patients with antibody deficiency disorders. The majority (90%) reported that they were not too familiar with SCIG; however, this is not unexpected, considering SCIG was only granted approval in the United States in 2006 (31) and its use among subspecialist immunologists still continues to expand. As such, 93% of family practice physicians who recommended Ig therapy for patients with PID indicated that they use primarily IVIG (primarily in an outpatient setting). Narrowing the gap in the prescribing patterns of Ig replacement and awareness of administration options may improve disease management for patients with PID.

### Use Vaccines in Patients with PID

**Figure 4:**
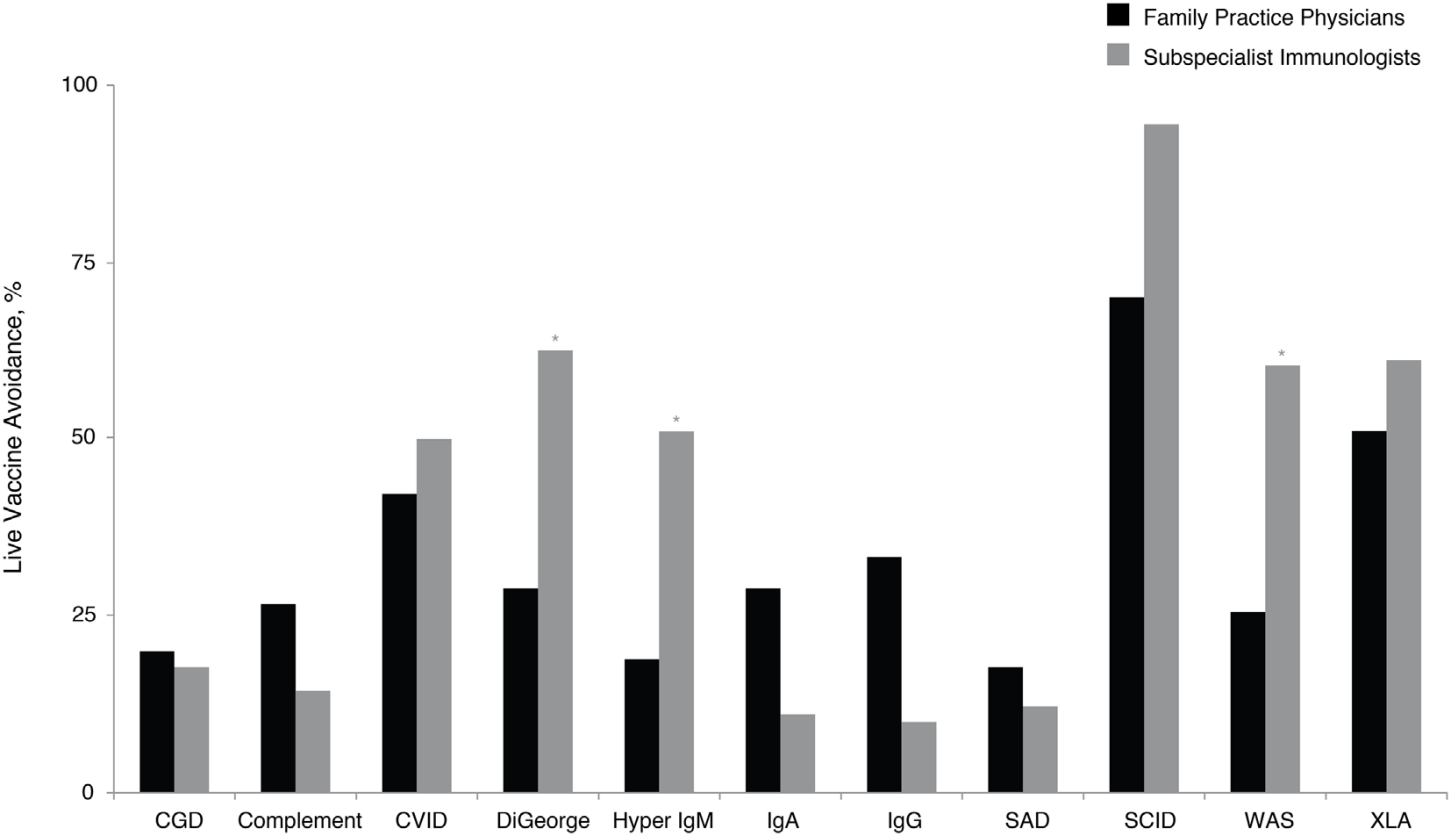
**Percentage of family practice physicians, compared with subspecialist immunologists, avoiding live vaccines for patients with specific PID**. *P < 0.0001. Ab, antibody; CGD, chronic granulomatous disease; CVID, common variable immunodeficiency; IgA, immunoglobulin A; IgG, immunoglobulin G; IgM, immunoglobulin M; PID, primary immunodeficiency disease; SAD, selective antibody deficiency; SCID, severe combined immunodeficiency; WAS, Wiskott– Aldrich syndrome; XLA, X-linked agammaglobulinemia (Bruton’s agammaglobulinemia).

Application of vaccines and the subsequent antibody response can indicate functional capacity of the immune system, and this assessment, although complex, can facilitate PID diagnosis. However, the use of live viral vaccines needs to be excluded in certain immunodeficiencies (14). These patients sometimes fail to produce a quality response to vaccines or are susceptible to harm from live vaccines (14, 32–35). Given the frequent use of vaccines by family practice physicians, this was viewed as an especially relevant topic. Family practice physicians recommended avoidance of live vaccines across a variety of PIDs (Figure 4). Significantly, more subspecialist immunologists than family practice physicians recommended avoidance of live viral vaccines for DiGeorge syndrome (61 vs. 26%, *P* < 0.05), hyper IgM (52 vs. 17%, *P* < 0.05), and Wiskott–Aldrich syndrome (57 vs. 23%, *P* < 0.05). There is an educational opportunity for family practice physicians regarding the potential risks of certain vaccines to patients with PID consistent with the prescriber information labels that contraindicate live viral vaccine use in PID, which could reduce the risk of complications in patients with certain PIDs.

## Discussion

This survey questioned family practice physicians practicing in the United States to assess their awareness of PID, along with the identification, diagnosis, and management of patients with these diseases. Evaluating routine practices and standards of care across a spectrum of physicians (e.g., pediatricians, family practice physicians, allergists, pulmonologists, and infectious disease specialists) who frequently encounter patients with PID may reveal opportunities in which dissemination of knowledge (e.g., PID recognition and diagnosis) may be useful. For example, a recent survey of pediatricians revealed that while 77% followed at least one patient with PID, over one-third (35%) were uncomfortable with PID recognition and diagnosis (26). Recommendations targeting other specialists, including pulmonologists, pathologists, and gastroenterologists, have already been published to raise awareness of the signs of undiagnosed PID (20, 36, 37). Family practice physicians may likewise benefit from similar educational initiatives. Therefore, the results of the current survey were compared with survey responses from subspecialist immunologists (23) to determine which areas need to be highlighted when developing educational materials. Given the appropriately diverse clinical interests of family practitioners, however, perhaps the most important message of our study is the potential benefits in aligning with immunology consultants when the question of PID arises. Recognizing PID is perhaps the hardest part of the equation, and the fact that so many family practice physicians had knowledge of patients and the diagnoses is enabling. The ability to identify and collaborate with clinical immunologists should be able to provide important steps in ensuring best practices within this rare disease population.

In the current survey, family practice physicians were generally not completely comfortable identifying and diagnosing PID, even though they will likely encounter a range of PIDs over the course of their practice. Interestingly, only 44% reported that they had followed a patient with a PID diagnosis. Given the emerging prevalence data that indicate PIDs are more common than once believed (3, 4, 6, 27) along with estimates of how many new patients a family practice physician might encounter over the duration of their practice, responses regarding diagnoses followed likely suggest that there are undiagnosed patients.

For some of the more serious diseases, onset occurs during infancy or childhood (2). However, PIDs affect all age groups and are more common in older patients than originally believed (38–40). Given that some PIDs, including CVID, CGD, and complement deficiencies, can be present during adulthood (27), family practice physicians may need to recognize and diagnose a more adult and elderly patient population. Indeed, in a 2008 survey of 1250 PCPs, 400 (32%) of the overall respondents [144 of 250 (58%) pediatricians, 148 of 490 (30%) internists and 108 of 510 (21%) family practice physicians] indicated that they had diagnosed, treated, or referred a patient with PID within the previous 5 years (41).

Patients with PID often present with increased occurrence of severe, persistent, and recurrent infections, autoimmunity, inflammation, allergy, and malignancies, which are associated with suppressed or inadequate immune function (1, 2, 16). These diseases are chronic, lifelong conditions requiring ongoing treatment and management. In a national survey of more than 1500 patients with PID, only 30% reported that they did not experience any degree of limitation in work, play, or normal physical activity because of their health (42). Current activity limitation and health status rating are both strongly influenced by permanent functional impairments that can occur prior to diagnosis (42). Patients often suffer from a significant time lag between symptom onset and the time of PID diagnosis (43–46). For all PID diagnoses, the average time from symptom onset to diagnosis has been reported to be 12.4 years (19). In the period preceding diagnosis, 54% reported developing a permanent functional impairment including, but not limited to, a decline in lung function, digestive function, and hearing (24).

Prevention of serious infections with timely intervention can improve patient outcome and quality of life following initiation of treatment (47, 48). Although treatment of PID is usually guided by an immunologist, a small number of family practice physician respondents actively manage IgG treatment for any patient (*n* = 4). Only a minority (15%) of family practice physicians who utilize IVIG therapy believed it to be very effective in the treatment of patients with PID. Because of the small number of responses regarding treatment management, results of the current survey do not provide adequate information regarding the dosages and intervals of dosing being used in the clinic by family practice physicians. In PID cases for which a family practice physician is the primary treating physician, knowledge of the most current treatment guidelines would ensure that patients receive the proper standard of care.

Surveys of this nature are always subject to non-response bias. The high proportion of non-responders could have been influenced by the fact that PID is considered to be rare (4); however, response rates were similar to those of the subspecialist immunologists (23). In an effort to increase response rates, two survey mailings were done and a financial incentive was offered to those who responded. Responses were kept anonymous in an attempt to reduce responder bias. A minimal amount of response bias has been shown in relation to survey responses among physicians (49, 50). That said, this work represents the first national survey of family practice physicians with regards to PID and suggest numerous opportunities with which diagnosis and care can be enhanced.

The present survey underscores the opportunity to improve education and training initiatives during medical school and in residency. Because notable differences exist in the way family practice physicians and general or subspecialist immunologists diagnose and manage patients with PID, it is relevant to continue efforts to increase awareness surrounding PID. The survey provides rationale supporting further initiatives designed to increase awareness and knowledge about PID among family practice physicians. The ability to consider and recognize PID patients is a critical task, which offers tremendous opportunity for a family practitioner to improve expected patient outcomes. This can only be improved further and linked directly to best practices for patients through education and collaboration with subspecialists in this increasingly complex field.

## Author Contributions

JO, FS, MB, CS, and VH-T conceived and designed the study, interpreted the data, revised the manuscript critically for important intellectual content, approved the final version for publication, and agreed to be accountable for all aspects of the work, including relating to accuracy and integrity. In addition, CS acquired and analyzed the data.

## Conflict of Interest Statement

Dr. JO has received grants from CSL Behring and Baxter; has received personal fees from CSL Behring, Baxter, ASD, Walgreens, and Atlantic Research; and serves on the medical advisory council to the Immune Deficiency Foundation. Dr. VH-T is a speaker for CSL Behring, Baxter, and Meda, a spokesperson for Sanofi and Merck, an advisory board member for Baxter, Sanofi, and Merck, an attendant at IFIR, and a medical advisory board member for the Immune Deficiency Foundation. Dr. FS, Ms. MB, and Mr. CS have no conflicts to disclose.

## References

[1] Al-Herz W Bousfiha A Casanova JL Chapel H Conley ME Cunningham-Rundles C Primary immunodeficiency diseases: an update on the classification from the international union of immunological societies expert committee for primary immunodeficiency. Front Immunol (2011) 2:1–26. 10.3389/fimmu.2014.00162 22566844 PMC3342372

[2] Reust CE. Evaluation of primary immunodeficiency disease in children. Am Fam Physician (2013) 87:773–8. 10.3928/00904481-20110316-08 23939499

[3] Bousfiha AA Jeddane L Ailal F Al-Herz W Conley ME Cunningham-Rundles C A phenotypic approach for IUIS PID classification and diagnosis: guidelines for clinicians at the bedside. J Clin Immunol (2013) 33:1078–87. 10.1007/s10875-013-9901-6 23657403 PMC4083684

[4] Bousfiha AA Jeddane L Ailal F Benhsaien I Mahlaoui N Casanova J-L Primary immunodeficiency diseases worldwide: more common than generally thought. J Clin Immunol (2013) 33:1–7. 10.1007/s10875-012-9751-7 22847546

[5] Parvaneh N Casanova JL Notarangelo LD Conley ME Primary immunodeficiencies: a rapidly evolving story. J Allergy Clin Immunol (2012) 131:314–23. 10.1016/j.jaci.2012.11.051 23374262

[6] Boyle JM Buckley RH. Population prevalence of diagnosed primary immunodeficiency diseases in the United States. J Clin Immunol (2007) 27:497–502. 10.1007/s10875-007-9103-1 17577648

[7] de Vries E Clinical Working Party of the European Society for Immunodeficiencies (ESID). Patient-centred screening for primary immunodeficiency: a multi-stage diagnostic protocol designed for non-immunologists. Clin Exp Immunol (2006) 145:204–14. 10.1111/j.1365-2249.2006.03138.x 16879238 PMC1809674

[8] Lim MS Elenitoba-Johnson KS The molecular pathology of primary immunodeficiencies. J Mol Diagn (2004) 6:59–83. 10.1016/S1525-1578(10)60493-X 15096561 PMC1867474

[9] Lindegren ML Kobrynski L Rasmussen SA Moore CA Grosse SD Vanderford ML Applying public health strategies to primary immunodeficiency diseases: a potential approach to genetic disorders. MMWR Recomm Rep (2004) 53(RR–1):1–29. 14724556

[10] Morra M Geigenmuller U Curran J Rainville IR Brennan T Curtis J Genetic diagnosis of primary immune deficiencies. Immunol Allergy Clin North Am (2008) 28:387–412. 10.1016/j.iac.2008.01.004 18424339

[11] O’Gorman MR. Recent developments related to the laboratory diagnosis of primary immunodeficiency diseases. Curr Opin Pediatr (2008) 20:688–97. 10.1097/MOP.0b013e328316ec16 19005337

[12] Joshi AY Iyer VN Hagan JB St Sauver JL Boyce TG. Incidence and temporal trends of primary immunodeficiency: a population-based cohort study. Mayo Clin Proc (2009) 84:16–22. 10.1016/S0025-6196(11)60802-1 19121249 PMC2630110

[13] Bonilla FA Bernstein IL Khan DA Ballas ZK Chinen J Frank MM Practice parameter for the diagnosis and management of primary immunodeficiency. Ann Allergy Asthma Immunol (2005) 94(5 Suppl 1):S1–63. 10.1016/S1081-1206(10)61142-8 15945566

[14] Orange JS Ballow M Stiehm ER Ballas ZK Chinen J De La Morena M Use and interpretation of diagnostic vaccination in primary immunodeficiency: a working group report of the basic and clinical immunology interest section of the American Academy of Allergy, Asthma & Immunology. J Allergy Clin Immunol (2012) 130(3 Suppl):S1–24. 10.1016/j.jaci.2012.07.002 22935624

[15] Jeffery Modell Foundation. 10 Warning Signs of Primary Immunodeficiency (2013). Available from: http://downloads.info4pi.org/pdfs/General10WarningSignsFINAL.pdf; http://www.info4pi.org

[16] Immune Deficiency Foundation. Diagnostic and Clinical Care Guidelines for Primary Immunodeficiency Diseases. 2nd ed (2009). Available from: http://primaryimmune.org/wp-content/uploads/2011/04/IDF-Diagnostic-Clinical-Care-Guidelines-for-Primary-Immunodeficiency-Diseases-2nd-Edition.pdf

[17] European Society for Immunodeficiencies. Clinical Diagnostic Criteria for PID (2006). Available from: http://esid.org/Working-Parties/Clinical/Resources/Diagnostic-criteria-for-PID2

[18] Jesenak M Banovcin P Jesenakova B Babusikova E Pulmonary manifestations of primary immunodeficiency disorders in children. Front Pediatr (2014) 2:77 10.3389/fped.2014.00077 25121077 PMC4110629

[19] Immune Deficiency Foundation. Primary Immune Deficiency Diseases in America: The Third National Survey of Patients (2007). Available from: http://primaryimmune.org/idf-survey-research-center/idf-surveys/patient-surveys/

[20] Hong J Knutsen AP Pulmonary disease in primary immunodeficiency disorders. Pediatr Allergy Immun Pulm (2013) 26:57–67. 10.1089/ped.2013.0227

[21] Salzer U Warnatz K Peter HH Common variable immunodeficiency – an update. Arthritis Res Ther (2012) 14:223 10.1186/ar4032 23043756 PMC3580506

[22] Prasse A Kayser G Warnatz K. Common variable ­immunodeficiency-associated granulomatous and interstitial lung disease. Curr Opin Pulm Med (2013) 19:503–9. 10.1097/MCP.0b013e3283642c47 23880700

[23] Yong PL Boyle J Ballow M Boyle M Berger M Bleesing J Use of intravenous immunoglobulin and adjunctive therapies in the treatment of primary immunodeficiencies: a working group report of and study by the Primary Immunodeficiency Committee of the American Academy of Allergy Asthma and Immunology. Clin Immunol (2010) 135:255–63. 10.1016/j.clim.2009.10.003 19914873

[24] Immune Deficiency Foundation. Treatment Experiences and Preferences among Patients with Primary Immunodeficiency Diseases: National Survey of Patients (2008). Available from: http://primaryimmune.org/idf-survey-research-center/idf-surveys/treatment-surveys/

[25] Hernandez-Trujillo HS Chapel H Lo Re V III Notarangelo LD Gathmann B Grimbacher B Comparison of American and European practices in the management of patients with primary immunodeficiencies. Clin Exp Immunol (2012) 169:57–69. 10.1111/j.1365-2249.2012.04588.x 22670779 PMC3390474

[26] Hernandez-Trujillo VP Scalchunes C Hernandez-Trujillo HS Boyle J Williams P Boyle M Primary immunodeficiency diseases: an opportunity in pediatrics for improving patient outcomes. Clin Pediatr (Phila) (2015) 54(13):1265–75. 10.1177/0009922815574079 25780256

[27] Cooper MA Pommering TL Korányi K. Primary immunodeficiencies. Am Fam Physician (2003) 68:2001–8. 14655810

[28] Orange JS Hossny EM Weiler CR Ballow M Berger M Bonilla FA Use of intravenous immunoglobulin in human disease: a review of evidence by members of the Primary Immunodeficiency Committee of the American Academy of Allergy, Asthma and Immunology. J Allergy Clin Immunol (2006) 117(4 Suppl):S525–53. 10.1016/j.jaci.2006.01.015 16580469

[29] ABIM Foundation. Choosing Wisely: An Initiative of the ABIM Foundation (2014). Available from: http://choosingwisely.org/wp-content/uploads/2012/04/5things_12_factsheet_AAAAI.pdf

[30] Buckley RH. Immunoglobulin G subclass deficiency: fact or fancy? Curr Allergy Asthma Rep (2002) 2:356–60. 10.1007/s11882-002-0067-1 12165200

[31] Ochs HD Gupta S Kiessling P Nicolay U Berger M Subcutaneous IgG Study Group Safety and efficacy of self-administered subcutaneous immunoglobulin in patients with primary immunodeficiency diseases. J Clin Immunol (2006) 26:265–73. 10.1007/s10875-006-9021-7 16783465

[32] Levy O Orange JS Hibberd P Steinberg S LaRussa P Weinber A Disseminated varicella infection due to the vaccine strain of varicella-zoster virus, in a patient with a novel deficiency in natural killer T cells. J Infect Dis (2003) 188:948–53. 10.1086/378503 14513412

[33] Kroger AT Atkinson WL Marcuse EK Pickering LK Advisory Committee on Immunization Practices (ACIP) Centers for Disease Control and Prevention (CDC). General recommendations on immunization: recommendations of the Advisory Committee on Immunization Practices (ACIP). MMWR Recomm Rep (2006) 55(RR–15):1–48. 17136024

[34] Perez EE Bokszczanin A McDonald-McGinn D Zackai EH Sullivan KE. Safety of live viral vaccines in patients with chromosome 22q11.2 deletion syndrome (DiGeorge syndrome/velocardiofacial syndrome). Pediatrics (2003) 112:e325. 10.1542/peds.112.4.e325 14523220

[35] Mamishi S Shahmahmoudi S Tabatabaie H Teimourian S Pourakbari B Gheisari Y Novel BTK mutation presenting with vaccine-associated paralytic poliomyelitis. Eur J Pediatr (2008) 167:1335–8. 10.1007/s00431-008-0674-5 18317803

[36] Bright PD Rooney N Virgo PF Lock RJ Johnston SL Unsworth DJ. Laboratory clues to immunodeficiency; missed chances for early diagnosis? J Clin Pathol (2015) 68:1–5. 10.1136/jclinpath-2014-202618 25352642

[37] Costa-Carvalho BT Grumach AS Franco JL Espinosa-Rosales FJ Leiva LE King A Attending to warning signs of primary immunodeficiency diseases across the range of clinical practice. J Clin Immunol (2014) 34:10–22. 10.1007/s10875-013-9954-6 24241582 PMC3930833

[38] Carneiro-Sampaio M Moraes-Vasconcelos D Kokron CM Jacob CM Toledo-Barros M Dorna MB Primary immunodeficiency disease in different age groups: a report on 1,008 cases from a single Brazilian reference center. J Clin Immunol (2013) 33:716–24. 10.1007/s10875-013-9865-6 23354909

[39] Stein MR Koterba A Rodden L Berger M Safety and efficacy of home-based subcutaneous immunoglobulin G in elderly patients with primary immunodeficiency disease. Postgrad Med (2011) 123:186–93. 10.3810/pgm.2011.09.2474 21904101

[40] Verma N Thaventhiran A Gathmann B ESID Registry Working Party Thaventhiran J Grimbacher B Therapeutic management of primary immunodeficiency in older patients. Drugs Aging (2013) 30:503–12. 10.1007/s40266-013-0079-7 23605785

[41] Waltenburg R Kobrynski L Reyes M Bowen S Khoury MJ. Primary immunodeficiency diseases: practice among primary care providers and awareness among the general public, United States, 2008. Genet Med (2010) 12:792–800. 10.1097/GIM.0b013e3181f3e2c9 20885331

[42] Immune Deficiency Foundation. Primary Immune Deficiency Diseases in America: The Second National Survey of Patients (2002). Available from: http://primaryimmune.org/idf-survey-research-center/idf-surveys/patient-surveys/

[43] Urschel S Kayikci L Wintergerst U Notheis G Jansson A Belohradsky BH. Common variable immunodeficiency disorders in children: delayed diagnosis despite typical clinical presentation. J Pediatr (2009) 154:888–94. 10.1016/j.jpeds.2008.12.020 19230900

[44] Litzman J Stikarovska D Pikulova Z Pavlik T Pesak S Thon V Change in referral diagnoses and diagnostic delay in hypogammaglobulinaemic patients during 28 years in a single referral centre. Int Arch Allergy Immunol (2010) 153:95–101. 10.1159/000301584 20357490

[45] Wood P UK Primary Immunodeficiency Network Primary antibody deficiencies: recognition, clinical diagnosis and referral of patients. Clin Med (2009) 9:595–9. 10.7861/clinmedicine.9-6-595 PMC4952305 20095309

[46] Wood P Stanworth S Burton J Jones A Peckham DG Green T Recognition, clinical diagnosis and management of patients with primary antibody deficiencies: a systematic review. Clin Exp Immunol (2007) 149:410–23. 10.1111/j.1365-2249.2007.03432.x 17565605 PMC2219316

[47] Berger M Murphy E Riley P Bergman G The VIRTUE Trial Investigators Improved quality of life, immunoglobulin G levels, and infection rates in patients with primary immunodeficiency diseases during self-treatment with subcutaneous immunoglobulin G. South Med J (2010) 103:856–63. 10.1097/SMJ.0b013e3181eba6ea 20689467

[48] Woroniecka M Ballow M. Office evaluation of children with recurrent infection. Pediatr Clin North Am (2000) 47:1211–24. 10.1016/S0031-3955(05)70268-6 11130993

[49] Cull W O’Connor KG Sharp S Tang SF. Response rates and response bias for 50 surveys of pediatricians. Health Serv Res (2005) 40:213–26. 10.1111/j.1475-6773.2005.00350.x 15663710 PMC1361134

[50] Kellerman SE Herold J Physician response to surveys: a review of the literature. Am J Prev Med (2001) 20:61–7. 10.1016/S0749-3797(00)00258-0 11137777

